# Effectiveness and cytotoxicity of two desensitizing agents: a dentin permeability measurement and dentin barrier testing in vitro study

**DOI:** 10.1186/s12903-022-02424-7

**Published:** 2022-09-10

**Authors:** Ruodan Jiang, Yongxiang Xu, Feilong Wang, Hong Lin

**Affiliations:** 1 Department of Dental Materials, Dental Medical Devices Testing Center, Peking University School and Hospital of Stomatology, No. 22, Zhongguancun South Avenue, Haidian District, Beijing, 100081 People’s Republic of China; 2 Department of Prosthodontics, Peking University School and Hospital of Stomatology & National Center of Stomatology, Beijing, People’s Republic of China; 3 National Center of Stomatology & National Clinical Research Center for Oral Diseases & National Engineering Research Center of Oral Biomaterials and Digital Medical Devices & Beijing Key Laboratory of Digital Stomatology & Research Center of Engineering and Technology for Computerized Dentistry Ministry of Health & NMPA Key Laboratory for Dental Materials, Beijing, People’s Republic of China

**Keywords:** Desensitizing agents, Dentin permeability, Dentin barrier cytotoxicity test, Dentinal fluid, Remineralization, Glutaraldehyde

## Abstract

**Background:**

When evaluating the efficacy and safety of various desensitizing products in vitro, their mechanism of action and clinical utility should be considered during test model selection. This study aimed to evaluate the effects of two desensitizers, an in-office use material and an at-home use material, on dentin specimen permeability, and their dentin barrier cytotoxicity with appropriate test models.

**Methods:**

Two materials, GLUMA desensitizer (GLU) containing glutaraldehyde and remineralizing and desensitizing gel (RD) containing sodium fluoride and fumed silica, were selected. Human dentin specimens were divided into three groups (n = 6): in groups 1 and 2, GLU was applied, and in group 3, RD was applied and immersed in artificial saliva (AS) for 24 h. Dentin specimen permeability before and after each treatment/post-treatment was measured using a hydraulic device under a pressure of 20 cm H_2_O. The perfusion fluid was deionized water, except in group 2 where 2% bovine serum albumin (BSA) was used. The representative specimens before and after treatment from each group were investigated using scanning electron microscopy. To measure cytotoxicity, test materials were applied to the occlusal surfaces of human dentin disks under which three-dimensional cell scaffolds were placed. After 24-h contact within the test device, cell viability was measured via 3-(4,5-dimethylthiazol-2-yl)-2,5-diphenyltetrazolium bromide (MTT) assays.

**Results:**

GLU significantly reduced the dentin permeability and occluded the dentinal tubules when 2% BSA was used as perfusion fluid. RD significantly reduced dentin permeability and occluded the tubules, but permeability rebounded after AS immersion. GLU significantly decreased cell viability, but RD was non-cytotoxic.

**Conclusions:**

In vitro GLU application induced effective dentinal tubule occlusion only following the introduction of simulated dentinal fluid. RD provided effective tubule occlusion, but its full remineralization potential was not realized after a short period of immersion in AS. GLU may harm the pulp, whereas RD is sufficiently biocompatible.

## Background

Dentin hypersensitivity (DH) is a transient and sharp pain experienced when exposed dentin encounters thermal, mechanical, or chemical stimuli [1]. The prevalence of DH can reach over 90% depending on the population and methodology used [2–4]. According to the widely accepted “hydrodynamic theory” [5], the main factor causing DH is dentin permeability. Therefore, treatment strategies include sealing of the dentinal tubules to reduce dentin permeability and the activity of pulp nerves [6]. Dental materials for the occlusion of open dentinal tubules include inorganic fillers, polymers, protein denaturing materials, and tooth remineralization materials [7–9], which are marketed as in-office or at-home use products. With the continuous introduction of novel desensitizing agents, it is particularly important to evaluate their effectiveness and biocompatibility using appropriate in vitro models.

Scanning electron microscopy (SEM) can be used to directly observe dentinal tubule occlusion; however, the sealing shown on the dentin surface morphology does not necessarily lead to reduced permeability. The deposits produced by desensitizers may not be solid [10]. Moreover, materials that denature protein, such as glutaraldehyde, act on the dentinal fluid, which may not be observed in vitro [8]. Some studies have shown in SEM images that glutaraldehyde-containing desensitizers occlude the orifices of dentinal tubules [11, 12]. However, Pereira et al. indicated that glutaraldehyde-containing desensitizers did not produce particle precipitation at the opening or within the tubules as glutaraldehyde reacts with the plasma proteins present in the dentinal fluid [13]. Furthermore, the dentinal fluid may no longer remain after cutting and rinsing the dentin specimens in an in vitro test [14].

The evaluation of dentin permeability can directly reflect the occlusive effect of certain materials over dentinal tubules; hydraulic conductance is the main in vitro method of evaluation [6, 15]. Some studies have applied this method and designed different treatment procedures to simulate the oral environment, including its inherent chemical and mechanical challenges [8, 16–19], and others have attempted to introduce simulated dentinal fluid into the dentin specimens [8, 14]. The International Organization for Standardization has not provided standards for the evaluation of desensitization efficacy. Thus, evaluation methods close to the product mechanism and clinical conditions are more reasonable and acceptable.

Dentin desensitizing agents are materials used in direct contact with the dentin; therefore, cytotoxic substances may reach the pulp through the dentinal tubules. To achieve therapeutic effects, the long-term and repetitive use of desensitizing agents is usually recommended; therefore, these products must have good biocompatibility. Dentin desensitizing products usually must be tested for cytotoxicity before market release. These products contain various chemical components, some highly cytotoxic, such as fluoride, glutaraldehyde, and 2-hydroxyethyl methacrylate (HEMA) [20–23]. Glutaraldehyde exerts its cytotoxic effect over a wide concentration range [23]. HEMA can inhibit the proliferation of epithelial cells and pulpal fibroblasts [20]. However, some materials containing these components have no toxic or acceptable effects on dental pulp in vivo [24, 25]. Traditional in vitro cytotoxicity testing combined with a monolayer cell culture cannot simulate the three-dimensional (3D) cell growth observed in vivo, which may explain the discrepancy between in vivo and in vitro study results.

The dentin barrier test evaluates the chemical toxicity to the pulp tissue of dental materials contacting the dentin by mimicking the contact process between the materials and teeth and can predict clinical behavior with a reasonable probability, which may therefore help replace animal experiments [20–22, 26, 27]. At present, this test is not widely used to evaluate desensitizers. Previous studies on dentin barrier models testing desensitizers’ cytotoxicity to cells in a monolayer culture revealed different degrees of cytotoxicity [20, 22].

There are some debatable results in the literature regarding glutaraldehyde-containing densensitizers, which have been shown to obtain effective dentinal tubule occlusion without the introduction of simulated dentinal fluid [11, 12]. Other studies immersed the dentin specimens in diluted bovine serum before applying the desensitizing treatment [28, 29], though this is not a realistic substitute for dentinal fluid due to bovine serum’s high viscosity [30]; it is necessary to evaluate the permeability of glutaraldehyde-containing desensitizers with a suitable perfusion fluid. Products that claim the ability of remineralization should demonstrate recrystallization during the desensitizing process, which can be partially detected by permeability and SEM. There are certain advantages to investigating the remineralization of desensitizers through the evaluation of permeability. The hydraulic pressure simulates the actual physiologic conditions of the body, so only the stable remineralized crystals can be maintained, thereby reducing dentin permeability. For both types of desensitizers, a dentin barrier cytotoxicity test was more applicable than the direct contact methods. Moreover, to our knowledge, no study has combined the dentin barrier test with 3D cell cultures to assess desensitizers.

Therefore, this in vitro study aimed to evaluate the effectiveness of two desensitizing agents (GLUMA desensitizer (GLU) and Remineralizing and Desensitizing gel (RD)), an in-office use material and an at-home use material, respectively, using hydraulic conductance and SEM observation, and evaluate the cytotoxicity of these two desensitizing agents through a dentin barrier test. Two different perfusion fluids, deionized water and 2% BSA, were chosen for GLU, and a post-treatment procedure of remineralization was chosen for RD. 3D cultures of transfected rat dental papilla-derived cells were used in the cytotoxicity test. The null hypotheses were: (1) application of GLU would be effective in reducing dentin permeability, whether deionized water or 2% BSA was used as the perfusion fluid, and the dentin specimens applied with RD would maintain the same permeability reduction after and before 24-h AS immersion; (2) neither GLU nor RD would demonstrate significant cytotoxicity in a dentin barrier cytotoxicity test.

## Methods

This study was approved by the Biomedical Ethics Committee of the Peking University School and Hospital of Stomatology (Process #PKUSSIRB-202060195). The authors stated that all methods were carried out in accordance with relevant guidelines and regulations. For the collection of isolated teeth, informed consent from patients was obtained.

### Test materials

Two desensitizers, GLUMA Desensitizer (GLU: Heraeus Kulzer, Hanau, Germany) and Remineralizing and Desensitizing gel (RD: American Hi Teeth Science and Technology Inc., USA), were used in this study and applied to the occlusal side of the dentin disks as per the manufacturers’ instructions. The details of all test and control materials are shown in Table 1.

**Table 1:** Tested desensitizing agents

Material	Manufacturer	Lot number	Main components
GLUMA desensitizer	Heraeus Kulzer GmbH, Hanau, Germany	52801	HEMA (36.1%), glutaraldehyde (5.1%), and purified water
Remineralizing and desensitizing gel	American Hi Teeth Science and Technology Inc., USA	31120011	Distilled water, glycerin, calcium carbonate, sodium bicarbonate, sorbitol, sodium fluoride, fumed silica, natural peppermint extract
Vitrebond (Positive control for the cytotoxicity test)	3M EPSE Dental Products	NC93061	Powder: glass powder and diphenyliodonium chloride Liquid: copolymer of acrylic and itaconic acid, water, and HEMA
Medical silicone (Negative control for the cytotoxicity test, Ф 6 mm × 2 mm)	Ji’nan Medical Silicone Rubber Products Factory	050701	Silicone rubber

### Tooth collection and dentin disk preparation

Thirty-eight extracted sound human third molars were obtained from patients aged 20–40 years. After removing debris and soft tissues, the teeth were soaked in 70% ethanol for 15 min [31] and stored in deionized water at 4 °C until use.

Dentin disks of 500 ± 50-μm were obtained by cutting the teeth perpendicular to their long axes, close to the pulp cavities [31], using a low-speed saw, (Isomet-Buehler, Lake Bluff, IL, USA) and all dentin disks were acid-etched with 35% phosphoric acid on both sides for 30 s each, using a previously described method [21, 32]. Each crown provided one disk, with an intact test area in each disk.

### Dentin permeability measurement

#### Specimen preparation

The dentin disks were randomly distributed into three groups (n = 6 in each group). Hydraulic conductance was measured for each acid-etched dentin disk and determined as the baseline permeability. In all groups except group 2, deionized water was used for perfusion.

*Group 1*: A small amount of GLU was applied to the acid-etched specimens and retained for 60 s. Subsequently, the specimens were air-dried, rinsed in deionized water, and measured immediately for permeability.

*Group 2*: The process was same as that for group 1; however, the perfusion fluid was replaced by 2% bovine serum albumin (BSA, Cohn Fraction V, pH 6.7; Kangyuan Biotechnology, Tianjin, China).

*Group 3*: RD gel was applied to acid-etched specimens and retained for 15 min. Subsequently, the specimens were rinsed in deionized water and measured immediately for permeability. These specimens were stored in artificial saliva (AS, Solarbio, Beijing, China) at 37 °C for 24 h and rinsed with deionized water; then, permeability was measured again.

AS was composed of deionized water, NaCl, KCl, Na_2_SO_4_, NH_4_Cl, CaCl_2_·2H_2_O, NaH_2_PO_4_·2H_2_O, CN_2_H_4_O, and NaF (pH 6.5). After the baseline permeability values were recorded, all treatments were conducted within 30 min. Representative SEM micrographs of the dentin disks were obtained.

#### Hydraulic conductance test

**Fig. 1:**
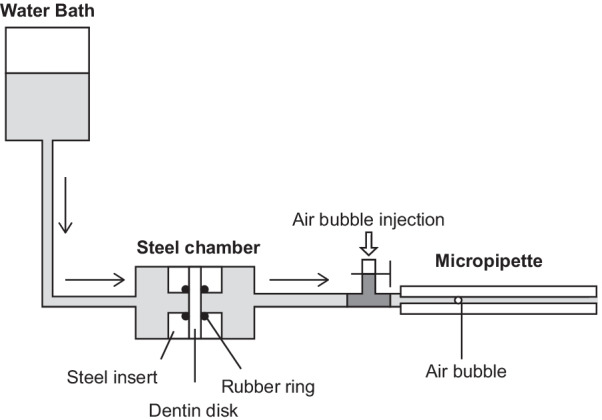
Dentin permeability testing device (hydraulic conductance device) used in the present study

The hydraulic conductance equipment was made in-house, as previously described [21, 32], according to the model designed by Outhwaite et al. and Pashley et al. [15, 33] (Fig. 1). The whole equipment was filled with perfusion fluid. The water bath provided a constant pressure of 20 cm H_2_O (1.96 kPa) to the pulp side of the dentin disk. A pair of rubber “O” rings limited the measurement area to 0.28 cm^2^ at the center of the dentin disk. One tiny air bubble was injected into a 100 µL-micropipette. The experiment was conducted after the air bubble kept moving steadily for 10 min. Each measurement was completed within 20 min. The minimum division value of the micropipette was 5 µL.

The volume of the perfusion fluid filtering through the dentin disk was measured by the linear displacement of the air bubble within a defined time period. Dentin permeability/hydraulic conductance, Lp (µl•min^−1^•cm^−2^•cm H_2_O^−1^), was calculated as:

$${\text{Lp}} = {\text{Jv}}/\left( {{\text{A }} \times {\text{ t }} \times {\text{ P}}} \right),$$

where Jv is the volume of fluid through the dentin disks (µL), A is the measurement area (cm^2^), t is the observation time (min), and P is the fluid pressure (cmH_2_O).

### SEM evaluation

SEM (EVO 18, Zeiss, Oberkochen, Germany) was used to observe the dentin specimens transversely and longitudinally before and after the desensitization treatments. Three dentin disks were prepared for each observation group. The specimens were dried in a desiccator for 48 h, fractured into two equal parts, sputter-coated with gold, and then observed under SEM at 10 kV or 20 kV at the selected magnifications of × 750, × 10,000 and × 5000.

### Dentin barrier cytotoxicity

#### Specimen preparation

Before the test, the hydraulic conductance of the dentin disks was assessed. The dentin disks with similar permeability were selected and divided into four groups (n = 5 in each group), ensuring that the mean value of permeability in each group was as close as possible. The four groups were randomly divided into two test and two control groups. The grouped dentin disks were used within three days.

During the cytotoxicity test, the smear layer on the occlusal sides of the dentin disks was reconstructed by grinding the occlusal sides of the dentin disks with 400-grit sandpaper at a consistent frequency and pressure for 15 s, and the dentin specimens were disinfected using 70% ethanol, as previously described [21, 32]. Concerning desensitizing agent application, GLU was applied on the occlusal surfaces of the dentin disks and kept for 60 s. Thereafter, the residual agent was air-dried. RD was applied on the occlusal surfaces of the dentin disks and retained for 15 min. The excess agent was wiped off using cotton swabs.

#### Cell culture

SV40 large T-antigen-transfected rat odontoblast-like cells, obtained from the rat dental papilla, were maintained in a minimum essential medium α medium (Gibco, USA) supplemented with 10% fetal bovine serum (Gibco, USA), 100 IU/mL penicillin, and 150 mg/mL streptomycin at 37 °C in 5% CO_2_ and 95% relative humidity. The 15th to 20th passage cells were used in this study.

The 8-mm diameter polystyrene 3D scaffolds (Nanjing Recongene, Nanjing, China), with four fiber layers, as previously described [21], were used for 3D cell culture. The scaffolds were placed in six-well culture plates and incubated for 48 h after adding 2 mL of cell suspension (1.5 × 10^5^ cells/mL). The cell-seeded scaffolds were moved to 24-well culture plates and cultured for 14 ± 2 d, changing the growth medium every other day.

#### Dentin barrier cytotoxicity testing

**Fig. 2:**
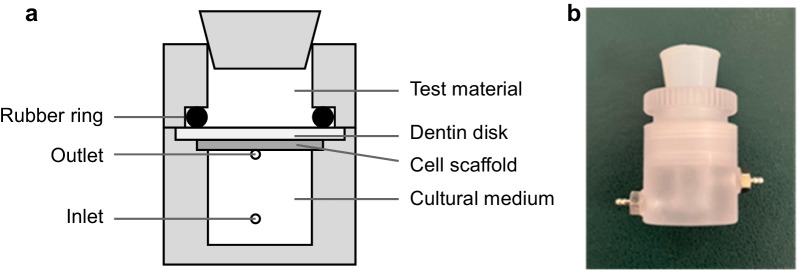
Diagram (**a**) and photograph (**b**) of the split chamber

The cell-seeded scaffolds were transferred into a 3D cell culture system (3D Biotek, New Jersey, USA) as previously described [21]. The main component, a polycarbonate split chamber, was a cylindrical cavity (Fig. 2). The dentin disk (occlusal side facing upward) was placed on top of the scaffold. The lower compartments of all split chambers were perfused with growth medium along with a 6 g/L HEPES buffer at 0.3 mL/h for 24 h at 37 °C. The liquid level of the growth medium covering the cell scaffolds was below the dentin disks. The test materials were applied and placed in contact with the dentin disks at 37 °C for 24 h after the perfusion was switched off [31].

The cell-seeded scaffolds were moved to a 24-well plate containing 0.5 mL MTT solution (Amresco, USA; 1 mg MTT/mL in PBS) and incubated for 2 h. Then, 250 μL of dimethyl sulfoxide was used to dissolve the formazan precipitate, and 200 μL of this solution was transferred into a 96-well plate, to determine the spectrophotometric absorbance at 540 nm.

### Statistical analysis

All statistical analyses were performed using SPSS software version 20.0 (SPSS, Chicago, IL, USA). For the dentin permeability measurement, Lp values were expressed as means and standard deviations, and the relative Lp values after treatments were expressed as percentages of baseline permeability values. The Shapiro–wilk test and Levene test were used to determine the normality and homoscedasticity of the data, respectively. The non-parametric Kruskal–Wallis test was applied to analyze the difference in baseline Lp values between the groups. The Friedman test was used to compare changes in Lp values before and after treatment/post-treatment within each group. A *P*-value < 0.05 was considered statistically significant. For dentin barrier cytotoxicity, results were expressed as percentages of the negative control. Statistical comparisons between groups were performed using the Mann–Whitney U test (α = 0.05).

## Results

### Dentin permeability measurements

The mean Lp values (± standard deviation [SD]) and their percent changes are shown in Table 2. The Kruskal–Wallis test revealed no significant differences in the baseline permeability between the three test groups (*P* = 0.172). The Friedman test showed a significant difference in the Lp values before and after treatment in group 2 (*P* = 0.014). Specifically, using 2% BSA as the perfusion fluid, dentin permeability decreased by 82% after GLU application. However, when deionized water was used as the perfusion fluid, dentin permeability in group 1 increased by 7% after GLU application, although the difference before and after treatment was insignificant (*P* = 1.000). In group 3, RD significantly reduced dentin permeability by 75% after 15 min of use (*P* = 0.014). After subsequent immersion in AS for 24 h, mean permeability rebounded by 28%, although there was no significant difference compared with the value before AS immersion (*P* = 1.000).

**Table 2:** Dentinal permeability measurements (Lp; mean ± s.d.) of different desensitizing agents, before and after treatments

Group	Permeability (Lp, µL•min^−1^•cm^−2^•cm H_2_O^−1^)			Relative Lp values (percentage of baseline Lp)		Perfusion fluid
	Baseline^†^	Treatments^‡^	Post-treatment (AS)^‡^	Treatments	Post-treatment (AS)	
Group 1 (n = 6)	0.155 ± 0.127	0.173 ± 0.161	n/a	107 ± 28	n/a	Deionized water
Group 2 (n = 6)	0.188 ± 0.132	0.043 ± 0.049	n/a	18 ± 17	n/a	2% bovine serum albumin
Group 3 (n = 6)	0.279 ± 0.163	0.081 ± 0.066	0.110 ± 0.088	25 ± 17	53 ± 42	Deionized water

All values are expressed as means ± standard deviations

^†^For each specimen, the mean of two measurements was taken

^‡^For each specimen, the first measurement data within 20 min was taken

### SEM evaluation

**Fig. 3:**
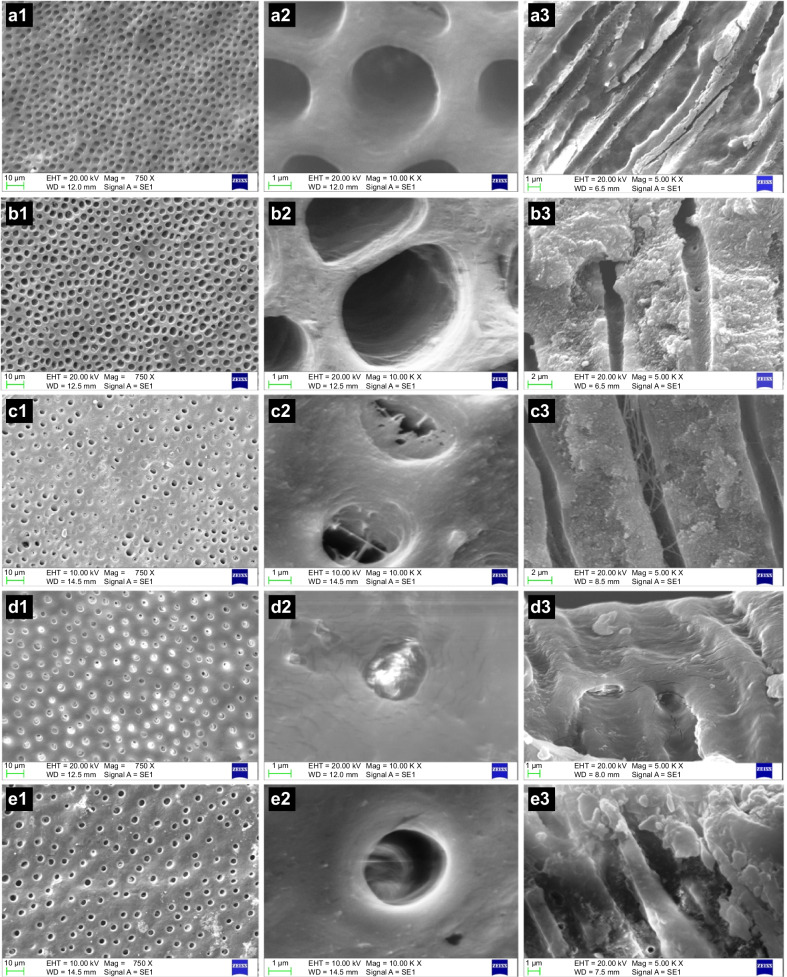
SEM images of dentin specimen surfaces and longitudinal sections before and after treatments/post-treatment. Magnifications: × 750 (left), × 10,000 (middle) and × 5000 (right). **a1**–**a3** SEM image obtained after acid etching in 35% phosphoric acid for 30 s, showing that the dentinal tubules are completely open and that the inside of the tubules is empty. **b1**–**b3** SEM image obtained after GLU treatment in group 1, showing that the dentinal tubules are completely open and that the collagen mesh of the demineralized dentin presumably has a certain degree of crosslinking by glutaraldehyde, but has not collapsed. **c1**–**c3** SEM image obtained after GLU treatment in group 2, showing that, under the effect of glutaraldehyde, nearly half of the tubule orifices are occluded due to the precipitation of the serum albumin remaining in tubules. Multiple reticular septa are observed in the lumen of the dentinal tubules. **d1**–**d3** SEM image obtained after RD treatment in group 3, showing that most tubule orifices are occluded by deposits and that the diameter of the tubules is reduced. A small amount of granular deposit is observed on the wall of tubules. **e1**–**e3** SEM image obtained after post-treatment by 24-h AS immersion in group 3, showing that most tubule orifices are exposed and that the amount of deposit inside the tubules has reduced. Some crystalline substances are observed inside the tubules. GLU, GLUMA Desensitizer; RD, Remineralizing and Desensitizing gel; SEM, scanning electron microscopy

Figure 3 shows the SEM micrographs of the dentin specimen surfaces and longitudinal sections of the three groups. Almost all dentinal tubules were open after etching, indicating that the smear layer and plugs had been removed (Fig. 3a1, a2). The dentinal tubules were almost empty (Fig. 3a3).

Figure 3b1–b3 shows the SEM images of the dentin specimens after GLU treatment in group 1. The surface morphology was similar to that after etching; most dentinal tubules were open, clear, and free of debris. Under the action of glutaraldehyde, the collagen mesh of the demineralized dentin seemed to cross-link to a certain depth but did not collapse (Fig. 3b3).

Figure 3c1–c3 shows SEM images of the dentin specimens after GLU treatment in group 2. Nearly half of the tubule orifices were blocked (Fig. 3c1). Since 2% BSA was the perfusion fluid in group 2, the serum albumin remaining in the tubules was probably precipitated by glutaraldehyde, which occluded the orifices and the interior of the dentinal tubules (Fig. 3c2, c3). Multiple septa were observed at a certain depth in the lumen of the dentinal tubules, and the reticular-like septa were in contact with the tubular walls (Fig. 3c3).

Figure 3d1–d3 presents SEM images of the dentin specimens after RD treatment, and Fig. 3e1–e3 presents SEM images after post-treatment by immersing in AS for 24 h in group 3. After RD treatment, the dentin surface was covered with a dense layer of deposit coating obtained from reaction products, and most tubule orifices were occluded (Fig. 3d1, d2). The tubules were evidently narrowed, and a small amount of granular deposit was found inside the tubules (Fig. 3d3). After post-treatment, many occluded tubule orifices reopened (Fig. 3e1, e2), and the amount of deposit inside the tubules decreased (Fig. 3e3) but more crystalline substances were observed inside the tubules (Fig. 3e3) than that in the specimens in Fig. 3d3.

### Dentin barrier cytotoxicity testing

**Fig. 4:**
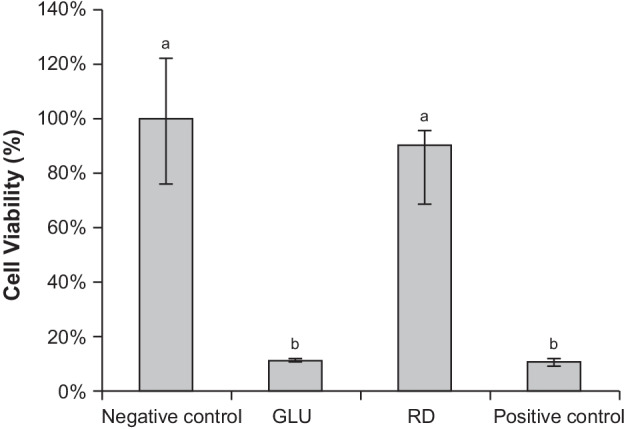
Cell viability of the 3D cultures of transfected rat odontoblast-like cells. Results are expressed as the percent cell viability relative to the negative control. The indicated values are the median and 25th and 75th percentiles. Different lower-case letters indicate statistically significant differences between groups (*P* < 0.01). GLU, GLUMA Desensitizer; RD, Remineralizing and Desensitizing gel

The test and statistical results are summarized in Fig. 4. GLU reduced cell viability to 11%, the result was not significantly different from that of the positive control (*P* = 0.310). Thus, GLU was severely cytotoxic. RD exhibited a cell viability of 90%, which was not significantly different from the result of the negative control (*P* = 0.421), showing the non-cytotoxicity.

## Discussion

Based on the results, GLU significantly reduced the dentin permeability only when 2% BSA was used as perfusion fluid. RD significantly reduced dentin permeability, but the permeability rebounded after AS immersion. Thus, the first hypothesis was rejected. GLU significantly decreased cell viability and the second hypothesis was rejected.

The present in vitro study evaluated the effectiveness and dentin barrier cytotoxicity of two desensitizing agents, an in-office use material and an at-home use material, using experimental models closer to the principle of material action and actual use in vivo.

Dentin permeability measurements revealed that when the perfusion fluid was deionized water, GLU did not occlude the dentinal tubules or affect dentin permeability. However, GLU significantly decreased the dentin permeability and occluded the dentinal tubules when the perfusion fluid was replaced by 2% BSA. The two active components of GLU are glutaraldehyde and HEMA. According to the literature, glutaraldehyde precipitates serum albumin in the dentinal fluid, and the coagulated proteins can form protein plugs that close the dentinal tubules [34, 35]. This protein coagulation leads to HEMA polymerization, and HEMA can facilitate glutaraldehyde penetration up to a depth of 200 μm in the dentinal tubules [34, 35]. Thus, dentin permeability can be significantly reduced [36, 37].

In fact, the dentinal fluid in isolated teeth is probably lost during a number of procedures, including cutting, etching, immersion, and ultrasonic cleaning. This study confirmed this point. In group 1, GLU did not close the dentinal tubules. Although glutaraldehyde appeared to cross-link the collagen mesh (Fig. 3b3), it had no effect on reducing dentin permeability. A similar collagen mesh morphology of the demineralized dentin disk was observed in a previous study using 2.5% glutaraldehyde as a fixation fluid [32]. The dentinal fluid is an important component of the pulp-dentin complex [34]. When the dentin is exposed, the outflowing dentinal fluid contains a fraction of plasma proteins [30]. Albumin is the main protein component in the plasma and dentinal fluid [38]. According to the protein fraction in the plasma and dentinal fluid, 1:3 diluted bovine serum was used to simulate the dentinal fluid in some studies [28, 38], although Özok et al. suggested that it was not a realistic substitute for dentinal fluid because of the relatively large molecular weight of the substances in the serum fraction [30]. Substances in the serum components with a molecular weight > 100,000 Da, such as globulins and lipoproteins, can reduce dentin permeability [30]. According to the literature, 2% BSA can be used as a simulated dentinal fluid [8, 14]. The bovine albumin used herein has a molecular weight of 66,000 Da; therefore, it did not affect the baseline permeability of the dentin. Our results revealed no significant differences in the baseline permeability between the groups.

Furthermore, the introduction of simulated dentinal fluid into tubules is key. Due to the capillary structure of the dentinal tubules, it is difficult for fluid to completely penetrate the tubules of the whole dentin disk during a short-term immersion. Therefore, this study used simulated dentinal fluid as perfusion fluid. After a short balance period, the albumin solution permeated the dentinal tubules, and baseline permeability was measured.

Using SEM, this study was able to detect reticular septa in the tubular lumen (Fig. 3c3), similar to the finding of Schüpbach et al. [35], where tubular occlusions were observed at a depth of 200 μm following the in vivo application of GLU and teeth extraction, and the septa led to the complete closure of the tubular lumen. This is inconsistent with Ishihata et al.’s study, which showed that no septa were found inside the dentin after it was soaked in albumin and GLU was applied [8]. The results from group 1 contradict previous studies which found that glutaraldehyde-containing desensitizers occluded the dentinal tubules or reduced dentin permeability without the introduction of simulated dentinal fluid [11, 12, 39, 40].

The main active ingredients of RD are sodium fluoride and fumed silica. Fluoride can penetrate dental hard tissue and bind with calcium salt, forming calcium fluorapatite deposits, thus occluding the dentinal tubules or reducing their diameter while promoting dentin remineralization [41]. Fumed silica, an amorphous nanoparticle, has a small particle size and large specific surface area, leading to strong surface adsorbability, large surface energy, high chemical purity, and good dispersion performance [42]. Therefore, it can be effectively deposited on the dentin surface to block the tubules. In the present study, the dentin permeability after application of RD was significantly reduced, as verified by SEM. RD may have formed a dense layer of material on the surface of the dentin (Fig. 3d1, d2). Subsequently, permeability increased after 24-h AS immersion. Notably, SD from the relative Lp values (Table 2) after 24-h AS immersion was relatively high, illustrating that the range of permeability changes was wide. According to the data, after a 24-h AS immersion, the permeability of half of the dentin disks increased compared with that after RD use; however, that of the other half decreased.

AS is usually used for in vitro studies to simulate an in vivo situation to assess erosion and remineralization in studies on DH [16, 17]. Immersion in AS may solubilize or wash away most debris from the dentin surface [16, 43], as found in this study (Fig. 3e1, e2). However, deposits inside the tubules were still observed (Fig. 3e3). Furthermore, more crystalline precipitates were found inside the tubules than before AS immersion (Fig. 3d3, e3). Phosphate groups on the surface of the collagen matrix can induce mineralization [44]. According to the report by Besinis et al., with the help of AS containing ionic calcium and phosphate, nano-silica can play the role of nucleating minerals in the collagen of demineralized dentin, enhancing the binding of calcium phosphate compounds to the collagen mesh and thus promoting remineralization [45]. Nanoscale silica can provide greater adhesion to calcium phosphate due to its large surface area, thus enhancing the potential of remineralization [46]. However, a short AS immersion time such as 24 h, may be insufficient to ensure adequate remineralization [17]. In this study, for some dentin specimens, the remineralization process after AS immersion was insufficient to maintain or enhance the occlusion of the dentinal tubules. In addition to the time factor and AS erosion, this may be related to individual differences in dentin specimens, such as tubular density and diameter. Generally, AS erosion plays a leading role, which may explain the rebound in the average relative Lp values after AS immersion. The current results were in line with a related study that also included a 24 h AS immersion [16].

The human dentin has a barrier function that can stop substances from penetrating into the pulp [20–22, 26]. Regarding dentin barrier cytotoxicity, GLU significantly decreased cell viability; however, the opposite was true for RD. Based on the existing research [22, 34], the high cytotoxicity of GLU exhibited in a dentin barrier test could be attributed to HEMA. Scheffel et al. found that 2.5–10% glutaraldehyde caused no obvious damage to odontoblast-like cells in a dentin barrier test, while glutaraldehyde integrated with HEMA showed high cytotoxicity [22]. In this cytotoxicity test, glutaraldehyde could react with the collagen to reduce its own concentration, and HEMA could be partially absorbed by dentin and collagen [34]. However, HEMA polymerization could not be completely induced due to inadequate serum albumin levels. It could be speculated that the cell culture medium containing fetal bovine serum cannot be fully introduced into the dentinal tubules due to the insufficient pressure on the pulp side of the dentin disks in this test device. The residual HEMA was the cause of high cytotoxicity.

HEMA has high solubility and low molecular weight, which makes it penetrate the dentin more easily. Even a small concentration of HEMA can irreversibly inhibit the cultured cells [47]. HEMA may cause DNA damage and mutation and even changes in gene expression, resulting in apoptosis [48]. Moreover, Yu et al. reported that at a concentration > 2 mmol/L, HEMA can induce the accumulation of intracellular reactive oxygen species in vitro, inhibit the proliferation and differentiation of dental mesenchymal cells, and activate the intracellular NF-κB pathway to induce autophagy formation [49]. The high cytotoxicity of GLU in this study is consistent with that observed using a similar method [20, 22] and with the precautions in the product instructions that deep cavities or areas close to the dental pulp should be properly capped.

Sodium fluoride has been proved to be cytotoxic in acidic environments [50]. A high concentration of sodium fluoride inhibited the proliferation of human epithelial cells, with different types of cells responding differently to sodium fluoride [51, 52]. Dogan et al. proved that mouse fibroblast cells were more sensitive to sodium fluoride than human keratinocytes and osteogenic sarcoma cells; they did not survive in sodium fluoride at 10 mM [52]. In the current study, RD exhibited low cytotoxicity, possibly because of its occluding effect on the dentin. Colloid components occluded dentinal tubules and precipitated, making it difficult to penetrate dentin disks. This type of desensitizer usually exhibits strong cytotoxicity in conventional in vitro tests, such as the filter diffusion and extract tests, where the contact mode between the materials and cells, cell types, and cell growth state vary from those observed in vivo. A recent study showed that desensitizers containing sodium fluoride were highly cytotoxic to monolayer gingival fibroblast cells; however, as the authors noted,the results of direct contact between materials and cells can be obviously inconsistent with clinical findings [9]. The SV40 large T-antigen-transfected rat odontoblast-like cells used in this study were obtained from rat dental papilla. This transfected cell line can be stably subcultured, have similar physiological properties to those of the dental pulp tissue, and have odontoblastic properties [53]. Combined with dynamic culture status used to simulate the blood flow of pulp tissue in vivo, the 3D culture of transfected odontoblast-like cells can reproduce the physiological and morphological characteristics of pulp cells in vivo to a certain extent [21].

The current study is limited by insufficient time for remineralization and lack of pulpal pressure in the cytotoxicity test, which can be improved in future studies. Considerably more work will need to be done to measure various types of densenitizers using test protocols that more closely mimic in vivo conditions.

## Conclusions

Our results revealed that GLU significantly reduced the permeability of dentin disks and occluded the dentinal tubules only when simulated dentinal fluid was used instead of deionized water as the perfusion fluid, indicating that the permeability evaluation of glutaraldehyde-containing desensitizers should use simulated dentinal fluid as perfusion fluid or other equivalent methods. RD significantly reduced dentin permeability and occluded the dentinal tubules, while this effect was decreased after 24-h AS immersion. The simulation of remineralization, such as post-treatment with AS, may be necessary in the evaluation of dentinal permeability reduction by desensitizers claiming to have remineralization potential. In this dentin barrier cytotoxicity test, GLU exhibited a significant cytotoxic effect, but RD was non-cytotoxic. GLU is recommended for use after pulp capping in deep cavities. RD, as an at-home use product, is safer than GLU.

## Data Availability

The datasets used and/or analysed during the current study are available from the corresponding author on reasonable request.

## Abbreviations

3D: Three-dimensional
AS: Artifical saliva
BSA: Bovine serum albumin
DH: Dentin hypersensitivity
GLU: GLUMA sensitizer
HEMA: 2-Hydroxyethyl methacrylate
MTT: 3-(4,5-Dimethylthiazol-2-yl)-2,5-diphenyltetrazolium bromide
RD: Remineralizing and Desensitizing gel
SD: Standard deviation
SEM: Scanning electron microscopy
